# How should community health workers in fragile contexts be supported: qualitative evidence from Sierra Leone, Liberia and Democratic Republic of Congo

**DOI:** 10.1186/s12960-020-00494-8

**Published:** 2020-08-08

**Authors:** Joanna Raven, Haja Wurie, Ayesha Idriss, Abdulai Jawo Bah, Amuda Baba, Gartee Nallo, Karsor K. Kollie, Laura Dean, Rosie Steege, Tim Martineau, Sally Theobald

**Affiliations:** 1 Department of International Public Health, Liverpool School of Tropical Medicine, Pembroke Place, Liverpool, L3 5QA UK; 2 College of Medicine and Allied Health Sciences, University of Sierra Leone, Freetown, Sierra Leone; 3 Institut Panafricain de Santé Communautaire et Medecine Tropicale, Bunia, Ituri Province Democratic Republic of Congo; 4 University of Liberia Pacific Institute for Research and Evaluation, Monrovia, Liberia; 5 Neglected Tropical Disease Program, Liberia Ministry of Health, Monrovia, Liberia

**Keywords:** Community health workers, Management support, Fragile settings

## Abstract

**Background:**

Community health workers (CHWs) are critical players in fragile settings, where staff shortages are particularly acute, health indicators are poor and progress towards Universal Health Coverage is slow. Like other health workers, CHWs need support to contribute effectively to health programmes and promote health equity. Yet the evidence base of what kind of support works best is weak. We present evidence from three fragile settings—Sierra Leone, Liberia and Democratic Republic of Congo on managing CHWs, and synthesise recommendations for best approaches to support this critical cadre.

**Methods:**

We used a qualitative study design to explore how CHWs are managed, the challenges they face and potential solutions. We conducted interviews with decision makers and managers (*n* = 37), life history interviews with CHWs (*n* = 15) and reviewed policy documents.

**Results:**

Fragility disrupts education of community members so that they may not have the literacy levels required for the CHW role. This has implications for the selection, role, training and performance of CHWs. Policy preferences about selection need discussion at the community level, so that they reflect community realities. CHWs’ scope of work is varied and may change over time, requiring ongoing training. The modular, local and mix of practical and classroom training approach worked well, helping to address gender and literacy challenges and developing a supportive cohort of CHWs. A package of supervision, community support, regular provision of supplies, performance rewards and regular remuneration is vital to retention and performance of CHWs. But there are challenges with supervision, scarcity of supplies, inadequate community recognition and unfulfilled promises about allowances. Clear communication about incentives with facility staff and communities is required as is their timely delivery.

**Conclusions:**

This is the first study that has explored the management of CHWs in fragile settings. CHWs’ interface role between communities and health systems is critical because of their embedded positionality and the trusting relationships they (often) have. Their challenges are aligned to those generally faced by CHWs but chronic fragility exacerbates them and requires innovative problem solving to ensure that countries and communities are not left behind in reforming the way that CHWs are supported.

## Introduction

Progress on Universal Health Coverage (UHC) will not be equitable or effective without specific action in Fragile and conflict-affected settings (FCAS). While definitions and figures vary, some 2 billion people are estimated to live in FCAS [1]. The share of extreme poor living in FCAS is expected to rise from 17% of the global total today to over 66% by 2030 as a result of shocks such as epidemics, earthquakes and climate change [1]. In FCAS, access to equitable and quality health services is essential for rebuilding the social and economic fabric of countries [2]. However, health indicators are especially challenging in FCAS compared with the regional and global averages (Table 1). For example, over 60% of the world’s child and maternal deaths occur in these settings [5].

**Table 1:** Health indicators in the 3 study countries, Africa region and Global (2019)

	Sierra Leone	Liberia	Democratic Republic of Congo	Africa region	Global
Maternal mortality ratio (per 100 000 live births)	1360	725	693	525	211
Under 5 mortality (per 1000 live births)	110.5	74.7	91.1	75.9	38.6
Neonatal morality rate (per 1000 live births)	33.5	25.1	28.9	27.2	17.7
Incidence of TB (per 100 000 population)	301.0	308.0	377.0	237.0	134.0
UHC tracer index (0–100)	49.5	51.4	43.9	N/A	N/A
SDG Global Rank (out of 162 countries)	155	157	160	N/A	N/A

Sources: World Health Statistics data visualizations dashboard [3]: SDG index and dashboards 2018 [4]

The health workforce is a key component of the health system that underpins the expansion of health services and UHC efforts. Most countries in the global South have a shortage of formal health workers and are increasingly looking to a range of community health workers (CHWs) to fill the gap and in particular reach the poorest and most marginalised communities. CHWs are arguably critical players in fragile settings, where human resource shortages are particularly acute as health workers may have been killed or fled during conflict or died during disease outbreaks such as Ebola.

Our three countries provide a unique opportunity to examine management of CHWs in fragile settings. In Sierra Leone and Liberia, the health systems were severely damaged by conflict and further weakened by the recent Ebola outbreak. Health systems’ responses during and post conflict emphasised the importance of CHWs’ understanding of their communities in the management of the outbreak as well as in re-establishing trust with the health system [6–8]. In both countries, the Ebola epidemic has triggered an increased interest and investment in community health programmes, with new policies recently rolled out [9, 10]. In Sierra Leone, a revised national Community Health Worker Policy was launched in February 2017 and rolled out nationwide with 15 000 CHWs trained to provide a basic package of services at the community level including reproductive, maternal, newborn and child health; integrated community case management of sick children; and infection prevention and control. In 2016, the Ministry of Health in Liberia launched its Revised National Community Health Services Policy 2016-2021. It focuses on the development of Community Health Assistants (CHAs—a type of CHW) trained to deliver a package of preventive, curative, promotive, rehabilitative and palliative services as well as surveillance. CHAs are supervised by Community Health Services Supervisors (CHSS)—a new cadre of health worker who have been formally trained, e.g. as a nurse. Previous cadres of community health volunteers (CHVs), for example, traditional midwives and community drug distributors, remain in operation and are supervised by CHAs.

Constant conflict, poor governance and infrastructure and an unfavourable business environment have left the DRC one of the poorest countries in the world, with the average Congolese resident living on less than $US 0.75 per day [11, 12]. The recent Ebola outbreak which has continued since 2018 has further weakened an already struggling health system [13]. DRC suffers from a severe shortage of health care personnel, with only 1.05 doctors, nurses and midwives per 1000 population [14]. *Relais Communautaires* or CHWs play an important role in providing health services to communities in insecure areas, and they are often the only health workers who stay. Supporting CHWs to continue providing services is an important issue. There is no overarching CHW programme in DRC, but CHWs are organised into three categories: site CHWs (providing a minimum package of community activities, such as distribution of ivermectin and contraceptives, case management of malaria, diarrhoea and respiratory infections); promotional CHWs (providing health education to communities); and disease-programme CHWs (providing specific services for the programme). We will use the term CHW to include CHWs in Sierra Leone and DRC and the CHAs in Liberia. None of these CHWs are salaried but instead receive allowances.

The role of CHWs in fragile contexts is emerging as critical, but the evidence base of what kind of support works best is still weak. Like other health workers, CHWs need support to ensure that they contribute effectively to health programmes, health system strengthening and UHC [15–19]. Management challenges, similar to those of managing other cadres of health worker, relate to improving attraction, retention and performance. While there is some literature on the application of individual human resource management (HRM) practices for CHWs in fragile settings [20–22], there is little evidence on the coordinated HRM approach to support CHWs, whereby HRM practices are designed to not only address expectations but also ensure that the CHW programme meets its goals [6, 23]. This paper will present qualitative evidence from three fragile settings on experiences of managing CHWs and synthesise recommendations for best approaches to support this critical cadre.

## Methods

### Study design

We used qualitative research methods to explore the management of CHWs in the three settings. This generated in-depth and contextual information about CHWs’, managers’ and decision makers’ experiences and perceptions as well as exploration of reasons behind their answers through probing questions [24, 25]. We used three methods: document review and key informant interviews with decision makers and managers, life history interviews with CHWs in Sierra Leone and document review and key informant interviews only in Liberia and DRC.

### Study settings

In Sierra Leone, two districts—Kenema and Bonthe—were selected following discussions between the research team and the CHW Hub in the Ministry of Health and Sanitation. Kenema is in the Eastern Province, is large with urban and rural areas and was heavily affected by the Ebola outbreak. Bonthe district is in the Southern Province, is hard to reach, riverine with several islands, and was less affected by the Ebola outbreak. We have worked in both districts before and have good working relationships with the District Health Management Teams. In Liberia, we selected two districts in Grand Bassa county: one district where international partners support CHW activities and one district where there is no current support for programme delivery. The research team has worked in this county before and has good relationships with the health management teams. We also conducted key informant interviews with national-level decision makers. In DRC, we worked in Ituri Province, a large province which is mainly rural and has experienced multiple inter-ethnic crises since 1999. As DRC is the second largest country in Africa and has decentralised health management to the provinces, we conducted the study at provincial level. Within Ituri province where our DRC co-author is based and has good relationships with the Provincial and District Health Offices, we selected two districts—Aru district (mainly rural) and Bunia district (urban).

### Data collection

Key informant interviews with decision makers and managers: Using country tailored topic guides, these interviews explored how CHWs are managed and supported. Decision makers and managers were purposively selected based on their involvement in developing community health policies, knowledge of community health programmes and managing CHWs. Table 2 provides an overview of the decision makers and managers included in the study in the three country settings. The research teams in each country conducted the interviews in the participants’ workplaces, in English (Liberia), French (DRC), and English or Krio (Sierra Leone), lasting between 40 and 90 min. They were recorded following consent of the participants.

**Table 2:** Participants for key informant interviews

	Sierra Leone	Liberia	DRC	Total
Decision makers at national and provincial level	4 (2F; 2M)	3 (3M)	2 (1F; 1M)	9
District-level managers	4 (1F; 3M)	3 (3M)	3 (1F; 2M)	10
Facility and community-level managers	11 (4F; 7M)	4 (2F; 2M)	3 (1F; 2M)	18
Total	19	10	8	37

*F* female, *M* male

Life history interviews with CHWs (Sierra Leone only): CHWs provided a personal account using their own words of their life and career over time [26, 27]. We selected CHWs from the two districts in Sierra Leone, ensuring that we had male and female CHWs, from different villages, and a range of ages and length of experience as a CHW (Table 3). CHWs drew a lifeline illustrating their life from birth until the present day, with an emphasis on their jobs and major life events. The researchers used this to explore their career with probing questions around becoming a CHW, their experiences as a CHW, support and human resource management strategies, relationships and interactions with existing cadres in the health system and informal workers and coping strategies. The interviews were conducted in Krio language and in private rooms in health facilities.

**Table 3:** Life history interview participants

	Gender		Age (years)			Experience (years)			Total
	Female	Male	20–29	30–39	40+	< 5	6–10	10+	
Kenema	4	4	2	3	3	5	3	0	8
Bonthe	5	2	1	3	3	1	5	1	7
Total	9	6	3	6	6	6	8	1	15

Document review: We reviewed key documents from the Ministries of Health in each country such as CHW policies, guidelines and training materials to answer key questions: What are the different types of CHWs? How are CHWs managed and supported in their work? What are the challenges to implementing CHW programmes? We extracted, summarised and synthesised text for each question.

### Analysis

**Fig. 1:**
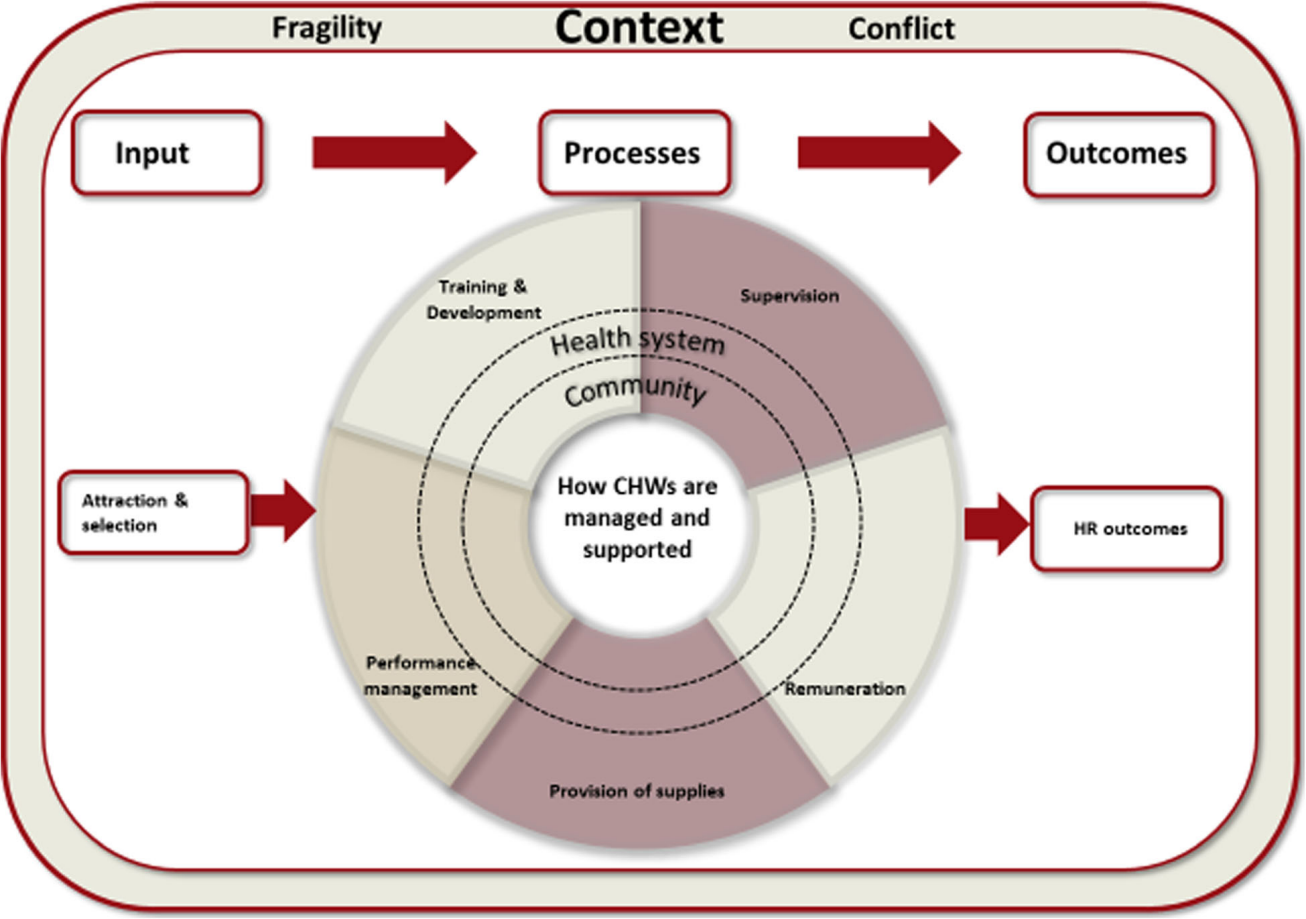
Framework to examine the human resource management of CHWs in fragile and conflict-affected settings

We transcribed all recordings verbatim and where necessary translated into English. We used the thematic framework approach to analyse the qualitative and document review data [28]. In the three settings, the teams reflected on the data as it was being collected and identified emerging themes for further exploration. We developed one coding framework (Table 4) for the three settings developed from the topic guides, research objectives, themes emerging from reading the transcripts and data and the HR and Community Health Worker frameworks (Fig. 1). The country teams applied the framework to the transcripts and data and developed charts for each code. The lead author then identified the initial themes through review of the initial analysis in each country and then shared these with the other authors to interrogate and refine. Consensus on key themes (e.g. HR outcomes, attraction, selection—gender and literacy, training and development, complex and challenging supervision, remuneration delays and repercussions, provision of supplies, challenges of rewarding and sanctioning volunteers) across contexts was reached through iterative reflection which involved the authors reviewing the themes, checking that the data supports the themes and adapting them. This was done by dialogue through e-mail and Skype and over a period of several months and consolidated in an analysis and writing workshop in Sierra Leone with all country teams present. By involving all authors, with different professional, personal and geographical backgrounds we ensured that different interpretations and perspectives were incorporated in the analysis [29, 30]. The qualitative analysis software, NVIVO, was used to help manage and analyse the data. These themes were discussed, and recommendations developed in a participatory workshop with CHWs, managers and decisions makers in Sierra Leone.

**Table 4:** Coding index

Code	Sub-codes
Range of CTC providers	
Reasons for becoming a CHW	
Role and responsibilities	Scope of work and services Workload and hours Number of CHWs Gender differences
Perceptions of being a CHW	Positive and negative Effects on family life Acceptance by community
Selection and recruitment	Selection process Effectiveness Challenges Recommendations Gender considerations
Attraction and retention	Strategies Effectiveness Recommendations Gender considerations
Provision of equipment, drugs, etc.	Ways that equipment, etc., supplied Effectiveness Recommendations Gender considerations
Supervision	Peer supervision Community supervision PHU supervision District-level supervision Central-level supervision Gender considerations
Training	Training needs Training provided Effectiveness Recommendations Gender considerations
Performance, rewards and sanctions	How CHW performance is assessed How CHWs are rewarded and sanctioned Effectiveness Recommendations Gender considerations Career pathway for CHWs
Monthly allowance	Amount and frequency Perceptions Recommendations
Community engagement	Community structures or people supporting CHWs Effectiveness of community engagement Recommendations
CHW integration into health system	Relationships with other health workers Views on integration Recommendations for better integration

## Results

We developed a framework to examine the HRM of CHWs in fragile settings (Fig. 1). This draws upon the CHW performance framework by Kok et al. [21] and the HRM approach defined by Armstrong [31] as “a strategic approach to acquiring, developing, managing, motivating and gaining the commitment of … the people who work in [the organisation] and for it” [page 33]. The HRM processes of attraction and selection, training and development, supervision, provision of supplies and performance management are influenced by the hardware (e.g. policies, guidelines, structures) and software (e.g. values and norms of the actors, relationships between the actors) of the community and health system and the broader context in which they exist. These influence the HR outcomes such as numbers and characteristics of CHWs and reported attrition. This framework provides the structure for reporting the results.

### HR outcomes

From the national decision makers and district managers, we found that there were fewer female CHWs in Sierra Leone and Liberia. In Sierra Leone, 14 935 CHWs were trained across the country: 10 652 males (71%) and 3283 females (29%). In Grand Bassa County, Liberia, there were 91 male (90%) and only ten female CHWs (10%). In DRC, district managers reported that in Bunia district, there were 480 CHWs, of whom 288 (60%) were female, and in Aru district out of 840 CHWs, 403 were female (48%). From the interviews with decision makers and managers in DRC, high attrition rates were reported, especially among younger and male CHWs who leave when they find better job opportunities. In Liberia and Sierra Leone, attrition issues were not reported by the decision makers, managers and CHWs probably as the community health policies have only recently been implemented.

### Attraction: wanting to serve their community

In Sierra Leone, most CHWs reported that they provided community health services before joining the new CHW programme, such as through being a traditional birth attendant or contact tracer during the Ebola outbreak. They wanted to continue to serve their communities and save the lives of pregnant women and children. In DRC and Liberia, decision makers and managers reported that CHWs were attracted to the role in the expectation of remuneration and wanting to serve their community.

### Selection: the tricky issues of literacy and gender

The Community Health Policies in Liberia and DRC include literacy as a requirement for being a CHW [9, 10]. In Sierra Leone, the CHW policy states that “*literacy and basic numeracy is highly valued and preferable, but is not strictly required, especially in the case of female candidates*” [9], p21]. Decision makers and managers in DRC and Liberia reported that it was challenging to find people with reading and writing skills who wanted this role. Decision makers and managers in Sierra Leone reported that some CHWs were unable to read or write which created problems with training, drug administration and reporting. Decision makers and managers in all settings and the CHWs in Sierra Leone explained that conflict disrupted education and this influenced the literacy and numeracy for some community members. Decision makers and managers in all settings recommended that basic literacy training is needed.

In Sierra Leone and Liberia, the policies state a preference for women, while in DRC, there are equal opportunities for men and women in the selection process [9, 10]. However, in all contexts, policy ideals were mediated in practice by gendered community norms. In both Sierra Leone and Liberia, there were more male CHWs. Interviews with managers and decision makers in Sierra Leone and Liberia explained that community-based selection processes, women’s limited voice and presence in community affairs, along with a culture of selecting men for paid work, emerged as reasons for more male CHWs. As one manager explained:

When it comes to community affairs, only men show up, women don’t, they don’t even talk. Should they show up, then the community members would have selected them*.* (District manager, male, Sierra Leone).

In DRC, the managers explained that there were more female CHWs where there were women’s associations. These associations advocate for women and influence husbands’ and relatives’ permission for women to join the programme.

*“*Here in our health district, where you find nearly half of community health workers are female, there are women associations, but where there are no women associations, you find that there are more male community health workers”. (District manager, male, DRC)

### Training and development—opportunities for learning and supporting each other

In all three settings, the policies state that the CHW role is to connect communities and the health system, providing basic health care services, health promotion, health surveillance and mobilising communities [9, 10]. The policies in all settings map out the training requirements for the CHWs (Table 5).

**Table 5:** Training of CHWs

	Sierra Leone	Liberia	DRC
Initial training	Standardised package that includes three modules: (1) Community health basics (e.g. communication, community entry, household registration, surveillance, health education, preventive care for children, identification of pregnant women); (2). Integrated community case management “plus” (e.g. assessment, referral, treatment and counselling, follow-up care for sick child or child with malnutrition; assess and treat adults with malaria; (3). Reproductive, maternal, newborn and child health (e.g. RMNCH continuum of care, family spacing, pregnancy visits, newborn visits, child visits). 6-8 days training for each module, face to face, with additional 1-2 days practical. No record of training evaluation.	Integrated and standardised training package includes modules on promotive, preventive and curative services, logistics, monitoring and surveillance. Each module is a month long with a 1-week face to face training delivered alongside implementation of services/practical experience. They must pass proficiency tests and supervision to progress to the next training module and function as a CHW. No record of training evaluation.	Site CHWs: 7 days’ training on treatment of common illnesses of children in the community such as simple malaria, diarrhoea, acute respiratory infections, and malnutrition. Promotion CHWs: 7 days training on health education and communication. Disease-programme CHWs: receive training specific to the programme. No description of mode of training or evaluation.
Refresher	Annual refresher training—no details in policy.	Twice a year training based on findings from supervision visits, and training needs assessments.	CHWs should receive refresher training, but no mention of frequency, duration or content.

In Sierra Leone and Liberia, initial training was done in modules over 1 year or 4 months, with each module focusing on different topics. Most managers and CHWs in both settings described the training positively, specifically the mix of classroom training and practical community experience, the provision of a manual, learning how to visit households, communication skills and developing a sense of a cohort amongst the CHWs. One participant in Liberia described: “In the evening, they will sit in group, ask one another questions… when we were in the training today, what you didn’t understand? - The person will explain, they all put their minds together”. (Facility manager, female, Liberia)

In Sierra Leone, CHWs reported that CHWs with limited literacy found it difficult to use the manual, make notes and review what was learned during the sessions. In Liberia, managers wanted to be more involved in the training so that they could understand what was expected of the new health cadres.

In DRC, training was reported to be more ad hoc. Managers explained that it was usually organised by the district health authorities when new CHWs were selected, or when there were national health campaigns. Managers reported that female CHWs were less likely to attend training because of their gendered responsibilities within families.

### Supervision—complex and challenging

Supervision of CHWs is a complex process and is the responsibility of a variety of different actors within the health system as reported in the policy documents (Table 6). There are some successes with this supervision but also significant challenges.

**Table 6:** Key actors involved in supervision of CHWs in Sierra Leone, Liberia and DRC

*Sierra Leone*	*Liberia*	*DRC*
Peer supervisor (CHWs with additional training): monthly visits to observe the CHWs work, check drug supplies and reports and coordinate monthly meeting of CHWs at Peripheral Health Unit.	CHSS: provides field-based supervision to 10 CHWs working in remote catchment communities, collates reports from CHWs and takes to the facility.	Chair of CHW group: organises monthly meetings, reports to the head nurse, who then reports to the District Health Office.
Peripheral health unit manager: regular visits to each CHW, attend monthly CHW meeting, provide advice and training to CHW, distribute drugs and supplies, compiles CHWs reports and sends to District CHW focal person.	Facility Manager: checks CHWs reports and clarify any issues, and report to the district health team.	Facility head nurse: regular visits to observe CHW work and records, provide training when needed such as implementing a specific programme or when a health problem increases.
District CHW focal person: provides training, visits the CHWs and the peer supervisors, collates reports from facilities and compiles district report for District Health Office and National Hub.		

In Sierra Leone, many CHWs were positive about the peer supervisors, reporting frequent contact and help with completing reports. This motivated them to visit households, to follow the guidance given during training and to contact the supervisors when there were problems that they could not solve. As one CHW explained:

Every week the peer supervisor visits. It wakes me up to do my work. (CHW, male, Sierra Leone)

Some CHWs in Sierra Leone valued the meetings at the local health facilities where they could discuss issues and collectively solve problems:

Well the meeting is good, it brings cordiality and makes the work easier because any issue you do not understand you can bring it up and they explain it to you. (CHW, male, Sierra Leone)

In DRC, facility managers reported linking their supervision visits to other activities and providing training and advice. They thought the supervision worked well in identifying where the CHWs were working well and any issues, and this supported the CHWs to provide services.

...for the supervision, we go on the ground, we see what they are doing … at the facilities level, there are other orientations we give them, for example organising census within the health catchment area. (Facility manager, female, DRC)

There were several challenges with supervision. Peer supervisors and the District CHW focal persons in Sierra Leone and the CHSS in Liberia reported no bicycles or transport allowance as a significant barrier to their work. They often travelled long distances, sometimes at their own cost. One CHSS explained the challenges she faced:

I have to walk on seven hours distance to go for supervision, and then I have to supervise the CHAs on two hours. Before I come back, darkness will catch me and I will sleep there. No compensation. I spend more time in the field, so they should see about compensating me for accommodation and feeding. These things can really affect performance. (Community manager, female, Liberia).

Workload was also cited by managers in all settings as a key reason for supervision structures not working effectively. As one key informant in Liberia explained: “In the policy it says that CHSS will visit the CHA twice a month. The reality is that, some of them have not been able to reach to the CHA to supervise them even once a month. This is because the CHSS must work 20% of their time in the clinic, but the clinic work takes up most of their time”. (National decision maker, male, Liberia)

In Sierra Leone, a few CHWs reported that the relationship between CHWs and health facility staff is strained in some areas: CHWs feel ignored, are not given drugs or supplies and are not selected for other community activities despite this being a good income source. One CHW reported being threatened by a manager: “He also told us that if he had known earlier, he would have removed our names from the programme because we’re not cooperative - we don’t give him any money from the incentive we are receiving.” (CHW, Sierra Leone, female). Some managers explained that some health facility staff see CHWs as taking their work (which provides income that supplements their sporadic salary) and being given drugs that are in short supply.

### Remuneration—delays and repercussions for retention and performance

In Sierra Leone and Liberia, the community health policies state that CHWs should be given allowances. In Sierra Leone, CHWS should receive 100 000 Leones plus 50 000 to 80 000 Leones for transport and other logistics per month (equivalent of US$18–24). The monthly salary for a nurse is approximately US$400. In Liberia, each CHW should be given US$70 per month which is based on provision of a package of health care at the household level through a minimum of 4 h work per day. The monthly salary for a nurse is approximately US$350. The policy also states that they may also receive other forms of motivation, such as transportation, gifts in-kind, employment and advancement opportunities, involvement in national campaigns and recognition events.

Most CHWs in Sierra Leone perceived the allowance to be too small to support themselves and their family and in relation to the amount of work they do.

We want them to increase our salary - pay us monthly and increase on the amount. Monthly payment is the best as we have personal commitments that require financial inputs and the amount is small, we want them to look into it critically ….and also they should take into consideration that the job is an everyday job and it involves us working with our community, it is very tedious and we even work at night when there are emergencies. (CHW, female, Sierra Leone)

In both settings, there were significant delays in CHWs receiving their allowances. In Sierra Leone, at the time of data analysis, district managers reported that CHWs had not received their allowance due to delays in setting up mobile phones and accounts to receive money. CHWs reported that they used their own money to travel around their community and attend meetings and training. Community members do not help CHWs with their farm work, and so CHWs have less time to do health work. In Liberia, managers reported that bureaucracy between the donor and the Ministry of Health has led to payment delays.

In DRC, CHWs are voluntary roles without remuneration. However, managers reported that the CHWs receive some financial compensation if they work for specific programmes, go on training, or from sales of health products such as bed nets. This money is irregular, and the amount varies per month, depending on their sales, the training and work opportunities and how much the programmes provide. They often have to use their own resources to visit households or attend meetings at facilities. Managers explained that despite being told about the voluntary nature of their work during the selection process, CHWs still expect to receive financial incentives. As this expectation is not met, they look for other work. Managers suggested that regular payment would motivate CHWs to work and reduce attrition.

...as they have to work voluntarily in context where finding a paying job is not easy. So, at the same they have to work for their survival and also for community. In a poverty context, their work is not easy. (District manager, male, DRC)

### Provision of supplies—promised but not always received

In Sierra Leone and Liberia, the community health policies emphasise the provision of adequate and quality assured medicines and supplies to treat uncomplicated malaria, acute respiratory infection and diarrhoea. In all three countries, managers and CHWs (Sierra Leone only) reported that challenges in the drug supply chain have led to delays in CHWs receiving medicines on time to treat patients, meaning their role has become predominantly to make referrals. In both Sierra Leone and Liberia, CHWs report to their supervisors when they run out of drugs or supplies, and then collect them from the local facility. Despite a proportion of drugs at the facility being allocated to CHWs, most drugs were used at facility level. In addition, Sierra Leonean CHWs reported spending their own money to travel to the health facility only to find either the drugs or the staff not there.

The distance we cover from our own community to the PHU, we go for drugs and drugs are not available, they will inform us that they haven’t received supply. (CHW, male, Sierra Leone)

CHWs and peer supervisors in Sierra Leone suggested that some drugs should be kept with the peer supervisors so that they can quickly “top up” the CHWs supply. In Liberia, managers recommended more rigorous and transparent process for allocation of drugs to CHWs is needed.

Despite promises of equipment and materials such as test kits for malaria, uniform, badge, torches, drugs boxes, thermometers, stationery and bicycles, CHWs in Sierra Leone and managers in all settings reported that most CHWs have not received these items. These are critical to CHW roles in promoting health and recognising and treating illnesses, making visits at night or in rainy weather and community recognition and trust.

CHWs need kits and identification card - some people will not speak to them or accept them without ID card, kits, rain boots, rain coats. (Community Manager, male, Sierra Leone)

### Performance management—the challenges of rewarding and sanctioning volunteers

In the three countries, there is no written guidance or indicators on how to manage or measure CHW performance. In practice, the facility managers reported that they assess CHW performance through their monthly reports of activities. In Sierra Leone, a health facility manager and peer supervisor gave each CHW a score based on the numbers and quality of household registrations and completed registers. Managers also reported that communities through the health facility committees play a role in monitoring CHW activities in Liberia, Sierra Leone and DRC. The committees review CHW reports, attend facility meetings and provide feedback from community members on the CHWs’ work. One manager in Sierra Leone explained how the committee identified a poorly performing CHW and worked with the managers to solve the problem:

They told me they cannot go to the man because he is always drunk so we have to change that CHW. (District manager, male, Sierra Leone)

Managers, in these resource poor settings, developed innovative ways to reward well-performing CHWs but found it difficult to sanction poorly performing ones (Table 7).

**Table 7:** Rewarding and sanctioning CHWs

	Strategies	Challenges
Rewarding CHWs “We think that the high performing CHWs should be recognised and awarded. This will make a big difference to how they feel appreciated”. (National decision maker, male, Liberia).	Selecting active CHWs for programme activities where they will be given a financial incentive	Not enough rewards and recognition
	Sharing food or small financial incentives during meetings	Create annual awards, certificates and radio announcement
	Providing verbal praise	Community recognition needs to be stronger in some areas: community members need to support CHWs with their farm work so that they can focus on their health work.
	Assuring CHWs that they have the community’s and God’s recognition	
Sanctioning CHWs “You know, it is not easy in our context to manage someone who works voluntarily, and does not benefit from financial incentives. It is just too difficult to objectively manage them”. (Facility manager, female, DRC).	More closely monitoring the CHWs and providing encouragement	Difficult to dismiss poorly performing CHWs
	Providing additional training and support	Time and resource consuming to replace CHWs
	Talking with the community to try to resolve performance problems	
	Occasionally, threatening not to submit the CHW report to the facility which would prevent them receiving their allowance.	

The managers in Liberia, DRC and Sierra Leone recognised the limitations of these rewards but perceived them to be valuable in supporting CHWs. As two managers explained:

As for rewards, we do not do something special, as we do not have money. But, whenever there is a meeting, we prepare food, and we eat together, and get to know each other much better. (Facility manager, female, DRC)

I didn't give them any reward, but the means…when a programme came, I didn't just let one keep working and working I started to rotate them when different programmes came and that allowed them to re-engage. (Facility manager, male, Liberia)

CHWs in Sierra Leone reported that the praise and recognition of the community is important in motivating them to continue their work. They wanted this recognition to be translated into more practical measures such as help with farm work and exemption from community tasks:

The community people sometimes give me words of moral boost and sometimes give me food items like fish, cassava. (CHW, male, Sierra Leone)

To make my job easier the community should at least assist me not financially but like if I want to make a cassava garden, they help me out, but they had not started doing that. (CHW, male, Sierra Leone)

## Discussion

This is the first study that has engaged with CHWs, managers and decision-makers and documented their views and experiences of the CHW programmes in the three fragile settings of Sierra Leone, Liberia and DRC. All settings have experienced and, in the case of DRC, are still enduring both conflict and disease outbreaks. All countries are now also responding to COVID-19 outbreaks, and the learning here can inform these responses. In Sierra Leone and Liberia, the 2015 Ebola outbreak brought the impetus for change in community health with new policies in both settings, with substantial financial and technical support from international partners. This is in sharp contrast with DRC where there have been no new community health reforms. Resources are scarce and trickle down from central level to provinces and districts. CHWs play a critical role in providing services to communities and linking communities to the health system in these settings. However, there are challenges to managing this cadre of health worker to ensure that they fulfil this role. Here we discuss these challenges and synthesise recommendations for best approaches to support this critical cadre, which are summarised in Table 8.

**Table 8:** Key findings and recommendations for management of CHWs in fragile contexts

	Attraction and selection	Training and development	Supervision	Remuneration	Provision of supplies	Performance management
Study findings	Literacy and gender played out in selection of CHWs. Fragility disrupts education of community members—CHWs may not have the literacy levels required for role; implications for selection, role, training and performance of CHWs. Selection policy ideals are mediated in practice by gendered community norms.	The modular, local and mix of practical and classroom teaching approach worked well in Sierra Leone and Liberia, helping to address gender and literacy challenges and served to develop a cohort of CHWs who support each other. Training in DRC is ad hoc.	Multiple actors involved in supervision. Peer supervision and some facility supervision seen as supportive. There are challenges with overloaded facility staff, limited transport, and limited support for supervisors. In Sierra Leone, relationships between facility health workers and CHWs are sometimes strained.	Delays in remuneration for CHWs in Sierra Leone and Liberia. CHWs use own money to do their work within contexts of poverty. Community think CHWs are paid and will not provide additional support. DRC CHWs still expect financial incentives, despite volunteer role.	Challenges in the drug supply chain have led to a delay in CHWs receiving medicines on time to treat patients, meaning their role is mainly to refer. Despite promises of equipment and materials most CHWs have not received these items. These are critical to CHW roles, reputation and community recognition and trust.	No written guidance on managing CHW performance. Managers use rewards, e.g. selecting active CHWs for programme activities, sharing food or small financial incentives during meetings, and providing verbal praise. Challenging to sanction poorly performing CHWs. Managers used encouragement, closer monitoring, additional training and support, and talking with the community to resolve performance problems.
Recommendations	Sensitise communities to encourage women to volunteer and to be selected at the same rates as men. Embed literacy training into CHW training to address literacy challenges. Support community development groups to create space for women’s active participation in community dialogue. Cultivate community “ownership” and support of CHWs from selection and throughout their ongoing role through regular meetings.	Provide training in a flexible, module-based approach with a mix of classroom and practical teaching; and app based training when travel is restricted. Learners can accumulate credits from modules, and pick up modules again if interrupted by conflict or other factors. Develop sense of a cohort so that CHWs support and learn from each other and jointly problem solve. Encourage mobile messaging or WhatsApp groups for ongoing peer support. Build ongoing capacity development needs into systems as CHWs roles may change e.g. during COVID19 pandemic.	Use innovative models e.g. peer supervisors, group supervision. Support the supervisors through training and recognition including in the provision of basic psycho-social support and strengthening CHW morale. Capture local issues and solutions to inform health system priorities. Encourage peer-to-peer discussions at routine CHW meetings at health facilities. Encourage community members to play greater role in support and supervision.	Clearly and openly communicate remuneration package with CHWs, other health workers and community. Develop robust system for timely payment and clearly communicate.	Provide drugs and other supplies on a regular basis. Ensure CHW supplies are allocated to CHWs by involving community and supervisor in allocation. Encourage sharing of resources within health system.	Reward good performance through recognition by peers and health system. Encourage community support and value. Develop a career pathway that reflects the needs of both female and male CHWs.

### Opportunities for selection

Conflict and fragility disrupts education of community members so that they may not have the literacy levels required for the CHW role, as seen in all contexts of this study. This has implications for the selection of CHWs, their role, training and performance. Policy preferences about selection need discussion at the community level, so that they reflect the realities of the communities. For example, community acceptability for certain services by a specific gender such as sexual and reproductive health [32–35]. To encourage selection of women, there is a need to sensitise communities to support and motivate women to volunteer and be selected at the same rates as men. When financial incentives are offered, communities select men who they deem more deserving of paid work, but are more likely to leave the role [36, 37]. Further, a lack of visibility of women in public limits their selection opportunities as demonstrated in Sierra Leone. Ensuring women’s active participation in community dialogue are particularly critical in fragile contexts and the creation of spaces where women are listened to and feel comfortable to talk and the development of further role models could support this. Women may also feel more empowered to volunteer when associated with community development programmes or women’s groups, as demonstrated in DRC.

### Changing roles of CHWs—the need for ongoing supportive training

CHWs are expected to undertake a wide range of activities including service delivery, health promotion and community mobilisation which reflects findings from other studies [6, 22, 38]. These areas will change over time as seen with the Ebola outbreak and the current COVID-19 pandemic. For CHWs to work effectively across these areas, substantial pre and in-service training is needed. This study shows that the modular, mix of classroom and practical teaching and locally based approach worked well in Sierra Leone and Liberia, which helped to develop a sense of a cohort of CHWs who support each other. Building a sense of camaraderie was also shown to be valuable to CHWs undergoing training in Mozambique [37]. Similar approaches should be applied for ongoing training—flexible, module-based approach close to CHWs homes to avoid long periods of time away from household responsibilities or by distance through mobile apps when travel is restricted. Modular-based training allows for CHWs to accumulate credits from individual modules and also take up modules again if interrupted by conflict or other factors. Encouraging peer support is critical for CHW retention and performance. Mechanisms include peer-to-peer discussions at the routine CHW meetings at the health facilities, mobile messaging and regular in-service training. In the study settings, a key role for CHWs is health surveillance. The new community health policies in Liberia and Sierra Leone were introduced after the 2015 Ebola outbreak, when preventing another outbreak was a national priority. This focus is reflected in the CHW training in Liberia and Sierra Leone, which emphasised household registration and monitoring for disease outbreaks. Critical to effective surveillance is the building of and maintaining trust so that communities are willing to disclose illnesses and seek care and do not perceive this as “spying”. This is particularly important in fragile contexts where many individuals and communities have faced trauma and resonates with the recent Ebola outbreak in DRC where CHWs played an important role in allaying fears about Ebola and supporting the Ebola vaccination campaigns [39].

### CHWs need a package of support

Training alone is not the panacea to effective community health programmes [6]. It is clear from this study that a package of supportive supervision, community support, regular provision of supplies, rewarding good performance and regular remuneration is vital to retention and performance of CHWs. But there are challenges with numbers of staff, limited transport and materials for supervision, scarcity of supplies at health facilities for the CHWs, inadequate recognition and support from some communities and unfulfilled promises about financial allowances. Supportive and responsive problem-solving supervision is critical. It should not be just top-down but capture local issues and solutions and inform health system priorities [40]. Innovations in supervision such as the peer supervisors in Sierra Leone and the CHSS in Liberia take the burden of supervision away from already stretched facility health workers. But for these cadres to really help CHWs, they need adequate support and recognition themselves.

Regular supply of drugs and materials is critical to the role and reputation of CHWs and for securing community recognition and trust. Health systems in fragile settings struggle with ensuring adequate supplies to facilities at all levels and in particular to remote areas because of limited finances for these commodities, weakened infrastructure and unsafe travel [41, 42]. Resources can also be a source of tension between CHWs and health workers as we have seen in Sierra Leone. Perhaps, this is a sign of a greater tension about how CHWs fit within the existing health system and how they work with facility health workers. Understanding this tension and openly talking about this would be a start to addressing this important issue.

Remuneration challenges have been a source of discontent amongst CHWs. CHWs should not be required to spend their own money and become impoverished through undertaking this role. Out of pocket payments by CHWs, linked to moral economies of care, add increased financial pressure to those least able to afford it and are not unique to these contexts [43–46]. Clear communication of the incentive package, as well as any delays is needed, not just with the CHWs but with other health workers and communities, so that they understand the constraints under which CHWs may be working. Irrespective of remuneration, community support—helping with farm work, providing transport and relieving them from other community duties—is needed [37]. Health system actors play an important role in encouraging community structures to support and value CHWs.

There are several limitations to the study. This study draws on qualitative methods and explores the issues from the perspectives of policy, CHW, decision makers and managers and does not reveal other important perspectives, such as those of the community and patients. The sample of CHWs was relatively small at 15, although this included a mix of genders, experience and settings. In DRC and Liberia, we did not include CHWs as this was designed as a rapid appraisal of the current situation in these two settings which included policy review and key staff knowledgeable about the CHW programmes. Further research that explores CHW direct experiences of management practices in these settings is needed. The participatory workshop in Sierra Leone enabled the findings to be validated with CHWs and key stakeholders in Sierra Leone and recommendations for managing CHWs to be developed grounded in the realities of fragile settings. Engagement of key stakeholders in this workshop illustrated their willingness to use research findings to adapt the policy and its implementation. Similar workshops in DRC and Liberia would be useful.

## Conclusion

In contexts of fragility and crisis, including disease outbreaks, CHWs interface role between communities and the health system is critical because of their embedded positionality and the trust they (often) have. Their role is further amplified due to severe human resource shortages particularly in rural areas. Common to all CHWs, they need support from an HRM perspective to make sure they can fulfil this role. CHWs, particularly in FCAS settings, have the most challenging of jobs and this is where HRM systems need to be built around them and respond to their particular evolving realities and contexts.

## Data Availability

The datasets are available from the corresponding author on reasonable request.

## Abbreviations

CHW: Community health worker
CHSS: Community Health Services Supervisors (CHSS)
DRC: Democratic Republic of Congo
FCAS: Fragile and conflict-affected settings
HR: Human resources
HRM: Human resource management
UHC: Universal Health Coverage
